# Towards integrated sustainability for China’s rural revitalization: an analysis of income inequality and public health

**DOI:** 10.3389/fpubh.2023.1328821

**Published:** 2024-01-08

**Authors:** Jie Lin, Kuiyuan Gong, Chuangbin Chen

**Affiliations:** ^1^School of Business, Shantou University, Shantou, Guangdong, China; ^2^Rural Revitalization Research Center, School of Business, Shantou University, Shantou, Guangdong, China

**Keywords:** China’s rural revitalization strategy, public health, rural development, income inequality, ordered logistic regression model

## Abstract

**Introduction:**

Ensuring healthy lives and promoting well-being are paramount among the priorities outlined in the 17 Sustainable Development Goals (SDGs) established by the United Nations. In China, rural revitalization stands as a pivotal national strategy aimed at fostering prosperity and sustainable development in rural areas. Despite its comprehensive evaluation system, which encompasses industry, ecology, culture, organization, and livelihood, the current index system overlooks the critical dimension of public health in rural areas. The existing body of literature predominantly focuses on the correlation between income and health, leaving a gap in understanding the relationship between income inequality and health from the perspective of villagers. This study addresses this gap by utilizing data from 3,771 villager samples and 302 village samples obtained from the 2019 China Rural Revitalization Survey (CRRS) to explore the correlation between income inequality and public health in China’s rural areas.

**Methods:**

We employ the Ordered Logistic Regression (Ologit) model in the baseline regression and heterogeneity analysis. Additionally, a mediating effect analysis, using the Sobel test, examines the role of villagers’ health awareness as a mediating variable in the correlation between income inequality and villagers’ health.

**Results:**

The empirical findings of this study unveil a statistically significant adverse influence of income inequality on public health in China’s rural areas. Furthermore, the research identifies that participation in regular exercise and the attainment of higher education levels serve as effective measures to alleviate the detrimental impact of income inequality on the health of rural residents. Additionally, income inequality is observed to shape villagers’ health awareness, thereby influencing their overall health status.

**Conclusion:**

The study’s outcomes have significant implications for policymakers and governmental authorities, providing valuable insights into some pathways for enhancing public health in rural China. Ultimately, these insights contribute to the broader objective of achieving integrated sustainability in rural China.

## 1 Introduction

The United Nations (UN) introduced the Sustainable Development Goals (SDGs) in 2015, outlining 17 goals, 169 targets, and 232 indicators to assess global progress in sustainable development. Among these, the third major goal (SDG 3) focuses on “Good Health and Well-being,” aiming to ensure healthy lives and promote well-being for all ages. SDG 3 has established a detailed set of scientific measures for individual and public health, bringing global attention to the importance of investing more in health-related issues. However, as developmental disparities continue to rise, achieving SDG 3 becomes more challenging (1). To enhance progress and achievements, the global SDGs agenda also highlights the significance of rural development. This emphasis is a crucial part of national strategies aimed at promoting a new initiative for rural revitalization (2, 3).

In China, rural residents account for 34.8% of the national total population, numbering 491.04 million (4). The Chinese Government introduced the rural revitalization strategy in 2017, driven by the need to address the growing material and cultural requirements of the rural population, particularly focusing on challenges associated with rural development (5). Aligned with the strategic plan for a sustainable economy (2018–2022), China has successfully achieved a stable grain output exceeding 0.65 trillion kg per year, with rural residents’ real *per capita* disposable income witnessing a substantial increase of 28.9 percent. The income disparity between urban and rural populations has decreased from 2.71 to 2.5, indicating noteworthy progress in economic sustainability. The comprehensive assessment system for rural revitalization includes five core goal dimensions: local economy, cultural enrichment, social governance, ecological sustainability, and overall well-being (6). Unfortunately, the rural revitalization strategy has not placed adequate emphasis on the critical aspect of public health. Given the substantial rural population in China, promoting the country’s rural revitalization strategy offers a practical reference for achieving global SDGs. Integrating the objective of enhancing public health into the overarching goal system of the current rural development process would enable a more comprehensive and integrated approach to achieving sustainability.

Health stands as a foundational human need, forming the cornerstone of individual well-being and family stability. The collective public health of a nation is instrumental in ensuring social stability and harmony. The significance of health has become increasingly apparent, particularly in the aftermath of the COVID-19 pandemic, which has imposed a growing financial burden on nations worldwide and disrupted global economies and health advancements (7). Given the substantial rural population in China, rural areas continue to grapple with challenges in public health, exerting a detrimental influence on the well-being and social satisfaction of rural residents (8). Consequently, this study is instituted with the aim of providing scientific insights to facilitate integrated sustainability, harmonizing economic development and public health. The research seeks to investigate potential impact factors on public health and the underlying mechanisms within the framework of China’s rural revitalization strategy.

The correlation between income and health has been extensively explored by scholars, supported by ample empirical evidence that highlights the significant impact of individual income on personal health. Notably, higher income levels are consistently linked to improved health outcomes (9). The influence of income extends beyond physical health, also manifesting in individuals’ life satisfaction and subjective well-being. This observation is underpinned by a nationwide analysis revealing a robust correlation between national income *per capita* and average life satisfaction (10). Countries with higher income levels generally exhibit a higher life satisfaction index compared to their lower-income counterparts. These studies on income and health span diverse samples, encompassing both developed and developing nations, establishing a well-documented relationship between these two variables (11–13). Given the well-established positive association between income and health, earlier scholars began to recognize the potential impact of income inequality on health, considering it as a pathway of influence (9, 14). Building upon collected baseline data encompassing county individual income, income inequality, self-rated health, and other variables, studies have indicated that individual income has a more pronounced effect on various health pathways, while income inequality appears to exert a modest influence on self-rated health but not on mortality (15). Recent research suggests that the impact of income on health is influenced by the interplay of both absolute income and relative income. Through decomposing their respective impacts and comparing their relative importance, the study found that relative income has a significant negative effect on health outcomes (16). This conclusion aligns with another study utilizing cross-sectional data from 7,070 participants in the Shandong Family Health Service Survey of Older People. Employing the binary logistic model and semi-parametric model, this research estimated the effect of absolute income and income relative deprivation (income inequality) on older people’s self-rated health. The results indicate that income relative deprivation is negatively associated with self-rated health in both urban and rural older populations (17).

In summary, the current research concerning income and health exhibits several insufficiencies. Firstly, while existing research has established and proven the linkage between income and health, there is a notable gap in deeper studies on income inequality and health. Many of the current studies lack clear distinctions in their selected samples, and even few have explored this relationship from the perspective of rural residents. To address this gap, our study aims to fill the gap in nuanced studies about income inequality and health, focusing on a group of Chinese villagers. Furthermore, China’s rural revitalization strategy has garnered increased attention in recent major government policies, positioning it as a central element in the construction of a modern, comprehensively developed country. However, the absence of a specific indicator to measure public health progress within this strategy has resulted in less public scrutiny on China’s rural public health. Concurrently, there is a scarcity of research focusing on the public health status in rural China. In an effort to address this research gap, our study employs the China Rural Revitalization Survey (CRRS) database from the Institute of Rural Development of the Chinese Academy of Social Sciences and aims to analyze how income inequality among Chinese villagers influences rural public health. This database is specifically designed to address rural revitalization-related issues and provides a more comprehensive review of China’s rural development including indices of income and health at both individual and village levels. Thus, compared to other general micro databases, the CRRS database provides more specific data for our current topic. Besides, our study employs various empirical methods to offer credible and scientific research findings with strong robustness and consistency. Utilizing data on the health status of sampled villagers and income inequality, this current study employs empirical methods such as OLS regression and Ordered Logit regression to evaluate the potential influence of income inequality on public health within the framework of China’s rural revitalization strategy. To ensure the robustness of our findings, the study conducts a Robustness test and Mediating effects analysis using the Oprobit model and Sobel test, respectively. These additional analyses aim to enhance the validity and reliability of research outcomes.

## 2 Materials and methods

### 2.1 Data sources and data processing

The data for this study were drawn from the China Rural Revitalization Survey, conducted by the Institute of Rural Development at the Chinese Academy of Social Sciences. Notably, the CRRS stands out for its recent and comprehensive dataset, offering insights into the economic and social dynamics of Chinese villages post the implementation of a rural revitalization strategy. Encompassing a broad survey sample across 10 provinces, including Guangdong, Zhejiang, Shandong, Anhui, Henan, Guizhou, Sichuan, Shanxi, Ningxia, and Heilongjiang, the study spans the eastern, central, western, and northeastern regions of China. Employing an equidistant random sampling method based on gross domestic product (GDP) *per capita*, the survey meticulously collected data at individual, household, and village levels. This included individual characteristics, family income, and village conditions. A total of 3,833 individual questionnaires and 308 village questionnaires were gathered. For analytical purposes, the study utilized the average income of the village to calculate the index of income inequality, assess villagers’ health conditions, and collect other relevant data to gage public health. Additionally, certain individual and village characteristics were incorporated as control variables. Following the exclusion of missing and anomalous data, our dataset comprises 3,771 villager samples and 302 village samples, forming the basis for the subsequent analysis.

### 2.2 Model specification

In order to investigate the relationship between income inequality and public health in China’s rural area, our empirical model is illustrated as follows:


(1) $$\mathrm{Healt}h_{i}=\alpha_{0}+\alpha_{1}RD_{i}+\beta_{1}X_{1i}+\beta_{2}X_{2j}+\varepsilon_{i}$$

where the dependent variable of $\mathrm{Healt}h_{i}$ denotes the health status of villager i ; the key independent variable of $RD_{i}$ is the index of income inequality of villager i ; $X_{1i}$ represents other personal characteristics that affect one’s health status, such as age, education, and exercise habits. In addition, considering some factors of villages may also influence local public health, such as locations and overall economic condition, we include control variables at the village level, which are denoted by $X_{2j}$ ; α and β are the influence coefficients of the corresponding variables; $\varepsilon_{i}$ is the error term.

Since the dependent variable in this study is an ordered variable, which is not statistically continuous, the method of Ordered Logistic Regression (Ologit) was adopted to estimate Eq. (1). The Ologit model is based on the cumulative distribution of the Logit model and assumes that the dependent variable is assigned ordinal values from 1 to J . The cumulative Logit for dependent variables ≤ *j* and >j can be expressed in Eq. (2).


(2) $$l_{j}\left(x_{j}\right)=\log\left[\frac{\mathrm{Pr}\left(y_{i}\leq j\;|\;x_{i}\right)}{\mathrm{Pr}\left(y_{i}>j\;|\;x_{i}\right)}\right]=a_{j}+\beta I+\varepsilon_{i}$$

where I includes all the independent variables and control variables and β is its coefficient. J represents the set of categories for grade ranking, $j\in J=\;\left\{1,2,3\right\}$ ; $\alpha_{j}$ represents the intercept term for Ologit estimation. Besides, the odds ratio of the Ologit model is calculated by the relative incidence ratio of x and y , indicating that when the independent variable increases by one unit, the dependent variable’s incidence ratio of the lower group is $e^{-\beta_{i}}$ times the incidence ratio of the adjacent higher group (18).

### 2.3 Variable measurement

#### 2.3.1 Dependent variable

The dependent variable of $\mathrm{Healt}h_{i}$ indicating villager’s health status is collected from the question in the villager’s questionnaire of CRRS: “How is your health status compared to your peers?,” and optional responses include “Very Bad,” “Bad,” “Average,” “Good,” and “Very Good.” We assigned a value of 1 to the variable of $\mathrm{Healt}h_{i}$ if the answer is either “Very bad” or “Bad,” a value of 2 if the answer is “Average,” and a value of 3 if the answer is “Good” or “Very Good.” Among the full samples, there are 522 villagers in poor health status (13.84%), 1,125 villagers in average health status (29.83%), and 2,124 villagers in good health status (56.32%) (Table 1).

**Table 1:** Descriptive statistics of variables.

Variable category	Variable	Variable description	Mean	SD
Dependent variable	Health	Health Status: Poor = 1; Average = 2; Good = 3	2.425	0.722
Key independent variable	RD	Relative Deprivation Index	0.508	0.269
Individual-level control variable	Exercise	Doing Exercise: No = 0; Yes = 1	0.580	0.494
	Age	The age of villager	54.919	11.876
	Gender	Female = 0 ; Male = 1	0.934	0.248
	Height	Logarithmic Height	5.107	0.048
	Weight	Logarithmic Weight	4.183	0.198
	Edu	Uneducated = 1; Elementary School = 2; Secondary School = 3; High School = 4; Technical Secondary School = 5; Vocational and Technical school = 6; Junior College = 7; Undergraduate = 8; Postgraduate = 9	2.760	1.082
	Information	Information Adequacy: Not at All = 1; Not enough = 2; Almost enough = 3; Enough = 4; Sufficient = 5	3.831	1.039
Village-level control variable	Distance	Distance to Town Government (km)	5.505	5.548
	Poverty	Identified as Village in Poverty: No = 0; Yes = 1	0.278	0.448

#### 2.3.2 Key independent variable

The key independent variable is the income inequality of villagers across China. The relative deprivation theory suggests that individuals are deprived when they compare themselves to people in their reference group and find themselves at a disadvantage. After grouping individuals based on their income levels, the lower the income of the individual, the greater the relative deprivation. Thus, relative deprivation is a concept at the individual level, whereas it is able to reveal the overall social distribution of incomes and income inequality. The Kakwani index has been widely applied in empirical studies to measure relative deprivation ( RD ) (17,19,20). In this study, the Kakwani index based on the villager’s total income in 2019 was adopted to represent the income inequality of villagers. Its calculation is as follows: Set X as a group, and the number of villagers in the group is n . By sorting the villager’s income in ascending order, we can acquire a total income distribution $X=\left(x_{1},x_{2},\cdots ,x_{n}\right),x_{1}\leq x_{2}\leq \cdots \leq x_{n}$ in Eq. (3).


(3) $$RD\left(x_{j},x_{i}\right)=\;\left\{\begin{array}{c} x_{j}-x_{i}\;\mathrm{if}\left(x_{j}>x_{i}\right)\\ 0\;\;\mathrm{if}\left(x_{j}\leq x_{i}\right) \end{array}\right.$$

The $RD\left(x_{j},x_{i}\right)$ of villager i implies the relative deprivation of $x_{j}$ to $x_{i}$ . Summing $RD\left(x_{j},x_{i}\right)$ over villager j and dividing by the villager’s income, we can acquire the relative deprivation of villager i in Eq. (4).


(4) $$\begin{array}{c} RD\left(x_{i}\right)=\frac{1}{n\mu_{x}}\overset{n}{\underset{j=1}{\sum }}RD\left(x_{j}-x_{i}\right)=\\ \frac{1}{n\mu_{x}}\left(\sum x_{j}>x_{i},x_{j}\in X^{x_{j}}-\sum x_{j}>x_{i},x_{j}\in X^{x_{i}}\right) \end{array}$$

Therefore, $RD\left(x_{i}\right)$ is a decreasing function of the villager’s income, and a larger $RD\left(x_{i}\right)$ indicates a higher level of village’s income inequality. $RD\left(x_{i}\right)$ has a range of values from 0 to 1. In addition, we also calculate $\mathrm{VillageR}D_{j}$ based on the average income of village j in 2019, which is used to replace $RD_{i}$ in the robustness test.

According to Zhang, the degree of income inequality can be classified into four levels based on the Kakwani index, namely Low $\left(\mathrm{Kakwani}<0.5\right)$ , Medium $\left(0.5\leq \mathrm{Kakwani}<0.668\right)$ , High $\left(0.668\leq \mathrm{Kakwani}<0.75\right)$ and Very high $\left(\mathrm{Kakwani}\geq 0.75\right)$ (21). As shown in Table 1, the average value of RD is 0.508, indicating that income inequality of villagers across China is at the medium level. We further calculated the average RD of the 10 sample provinces. Results show that Zhejiang Province has the lowest income inequality level of 0.315, while Shandong Province has the highest of 0.610. Other provinces have similar levels of income inequality, with RD of around 0.5.

#### 2.3.3 Control variables

There are two sets of control variables. Individual-level control variables include some personal characteristics, i.e., Age , Gender, Weight , Height , and other personal details influencing health, i.e., education status ( Edu ), exercise habit ( Exercise ), and sufficient online information ( Informaiton ). Specifically, the variable of Exercise corresponds to the CRRS question of “Have you done more than 30 min of exercises in the last week?.” The optional responses are “No” and “Yes” with assigned values of 0 and 1. The variable of Information refers to villagers’ ability to acquire online information, which corresponds to the question of “Do you think you have obtained sufficient information through the Internet for your daily life and work?” The optional responses are “Not at all,” “Not enough,” “Almost enough, “Enough,” and “Sufficient,” and they are assigned a value from 1 to 5, respectively.

Village-level control variables included village’s location and overall economic condition. Location is measured by its distance to town government ( Distance) . Overall economic condition is indicated by whether it is identified as village in poverty by the government ( Poverty ). The detailed description of variables and their statistics information are presented in Table 1.

## 3 Results

### 3.1 Baseline results

According to our empirical model, the relationship between income inequality and health is detailed in Table 2. Columns (1) and (2) present the estimated results using OLS regression. Notably, the coefficients of RD are consistently and significantly negative, even after incorporating a series of control variables. This underscores a substantial and negative correlation between income inequality and the health status of villagers.

**Table 2:** Baseline results.

Variable	(1)	(2)	(3)	(4)
	OLS		Ologit	
			Coefficient	Odds ratio
RD	−0.329^***^ (0.043)	−0.192^***^ (0.046)	−0.464^***^ (0.129)	0.629^***^ (0.081)
Exercise	0.126^***^ (0.024)	0.107^***^ (0.024)	0.313^***^ (0.065)	1.368^***^ (0.089)
Age		−0.003^***^ (0.001)	−0.008^***^ (0.003)	0.992^***^ (0.003)
Gender		0.0003 (0.048)	−0.007 (0.132)	0.993 (0.131)
Height		0.423 (0.273)	1.259^*^ (0.750)	3.522^*^ (2.641)
Weight		0.117^*^ (0.065)	0.313^*^ (0.186)	1.367^*^ (0.254)
Edu		0.030^***^ (0.011)	0.068^**^ (0.032)	1.071^**^ (0.034)
Information		0.054^***^ (0.012)	0.151^***^ (0.033)	1.163^***^ (0.039)
Distance		−0.003 (0.002)	−0.008 (0.006)	0.992 (0.006)
Poverty		−0.040 (0.026)	−0.096 (0.073)	0.908 (0.066)
Cons	2.519^***^ (0.029)	−0.285 (1.297)		
Numberofobs	3,771	3,771	3,771	3,771

Standard errors are in brackets; *, **, *** indicate significant at the significance level of 10, 5, and 1%, respectively.

We conducted the Ologit regression and found that the coefficient of RD is still negative at a significant level. The results show that with one unit decrease in income inequality, the odds of good health status versus the other two health status (average & poor) are 62.9% higher, provided that other variables remain constant. This verifies that reducing income inequality can effectively improve the public health in the rural area. Regarding the estimated results of control variables, Exercise , Weight , Height , Edu and Information exhibit significant positive influences on villagers’ health, while Age negatively affects their health. These outcomes align with logical expectations, affirming the rationale of our empirical model. However, the village-level control variables, specifically the village’s location and poverty condition, do not demonstrate significant influences on the health status of villagers.

### 3.2 Robustness test

In order to verify the robustness of the baseline model, we applied two methods, namely changing the regression model and replacing the independent variable (22). The results of robustness tests are shown in Table 3.

**Table 3:** Results of robustness test.

Variable	Replacing model	Replacing independent variable	
	(1)	(2)	(3)
	Oprobit model	Ologit model	Oprobit model
RD	−0.304^***^ (0.077)		
VillageRD		−0.455^**^ (0.205)	−0.322^***^ (0.123)
Exercise	0.182^***^ (0.039)	0.316^***^ (0.065)	0.183^***^ (0.039)
Individual Characteristics	Controlled	Controlled	Controlled
Village Characteristics	Controlled	Controlled	Controlled
Numberofobs	3,771	3,771	3,771

Standard errors are in brackets; *, **, *** indicate significant at the significance level of 10, 5, and 1%, respectively.

Initially, we replaced the Ologit model with the Ordered Probit Regression (Oprobit) model. The Oprobit model, while having similar applicability to the Ologit model, employs a different estimation method. If the results obtained from the Ologit model are robust, they should align consistently with the findings of the Oprobit model. As depicted in Column (1), the significantly negative effects of income inequality on villagers’ health persist, validating the robustness of our results.

Furthermore, we modified the former key independent variable, RD , to VillageRD . VillageRD is calculated based on the average income of the village in 2019, representing the income inequality among villages. Utilizing either the Ologit or the Oprobit model, the negative and significant correlation between income inequality and health is consistently maintained (Columns (2) and (3)).

### 3.3 Heterogeneity analysis

According to the baseline regression, Exercise and Edu are two important factors affecting villagers’ health. Thus, we used subsamples, namely villager groups identified by doing exercises or not and education level, to further explore the possible different effects of income inequality on public health in rural areas. In terms of doing exercises, we divided the full sample into two groups by the value of Exercise . In terms of the education level, the classification criterion is whether the villager obtains further education after the 9-year compulsory education (elementary and secondary school), and the two groups are named “Low education level” ( Edu≤3 ) and “High education level” ( Edu>3 ). The results of heterogeneity analysis applying Ologit regression are shown in Table 4.

**Table 4:** Results of heterogeneity analysis.

Variable	Doing Exercises		Not Doing Exercises		Low Education Level		High Education Level	
	Ologit							
	(1)	(2)	(3)	(4)	(5)	(6)	(7)	(8)
	Coefficient	Odds ratio	Coefficient	Odds ratio	Coefficient	Odds ratio	Coefficient	Odds ratio
RD	−0.614^***^ (0.171)	0.541^***^ (0.093)	−0.265 (0.197)	0.767 (0.151)	−0.542^***^ (0.140)	0.582^***^ (0.082)	−0.144 (0.331)	0.866 (0.287)
Exercise	−	−	−	−	0.320^***^ (0.070)	1.377^***^ (0.096)	0.340^*^ (0.180)	1.404^*^ (0.253)
Edu	0.084^**^ (0.042)	1.088^**^ (0.046)	0.044 (0.050)	1.045 (0.052)	−	−		−
Individual Characteristics	Controlled	Controlled	Controlled	Controlled	Controlled	Controlled	Controlled	Controlled
Village Characteristics	Controlled	Controlled	Controlled	Controlled	Controlled	Controlled	Controlled	Controlled
Numberofobs	2,189	2,189	1,582	1,582	3,205	3,205	566	566

(1) The group of Low education level includes those uneducated and only receiving compulsory education (elementary and secondary school); the group pf High education level includes those having further educations (high School, technical secondary school, vocational and technical school, junior college, undergraduate and postgraduate). (2) Standard errors are in brackets. *, **, *** indicate significant at the significance level of 10, 5, and 1%, respectively.

The results in Columns (1) and (3) indicate that there are apparent differences in the significance of RD . Among villagers doing exercises when income inequality is decreased by one unit, the odds of good health status versus the other two health status (average & poor) are 54.1% higher. The odds is smaller for the full sample in the baseline regression, indicating that doing exercise can eliminate the negative effects of income inequality on public health in the rural area. Hence, promoting the adoption of regular exercise among villagers emerges as an effective strategy to enhance people’s health, notwithstanding the challenges in preventing income inequality and its associated negative effects on public health. Notably, for villagers who do not engage in exercise, their health shows no significant correlation with income inequality, as they typically experience a decline in health status.

In our study, we found that the negative impact of income inequality on villagers’ health is significant only among those with a “Low education level.” Improving income equality can notably enhance the health of individuals with only compulsory education or less. On the other hand, for those with higher education, income inequality does not seem to affect their health negatively. This might be because well-educated villagers know more about how to take care of their health, especially through participating in social activities. This finding is similar to what another study suggested – that older people with higher education tend to feel healthier, have fewer depressive symptoms, face fewer daily activity limitations, and engage more in social activities (23). Therefore, it is a valuable practice by the government to promote education, especially in rural areas, which could contribute to better public health and overall living standards (24).

### 3.4 Mediating effect of health awareness

The previous regression and analysis confirmed the substantial and negative impact of income inequality on public health in rural areas. Now, we aim to delve into the crucial role of people’s health awareness in the connection between income inequality and villagers’ health. Dietary habits serve as a vital indicator of people’s health awareness and can profoundly affect both physical and psycho-social health, influencing overall life quality. Unhealthy dietary habits and lifestyles are widely acknowledged as significant risk factors for life-threatening diseases (25). Nonetheless, the precise mechanisms linking lifestyles and health outcomes remain unclear (26). Therefore, this study proposes to adopt a mediating effect analysis to examine the relationship between dietary habits, income inequality, and health levels of selected sample villagers. A mediating effect analysis based on the Sobel test is used. The Sobel test method has been found to have a strong detecting ability in the study of mediating effects (27, 28). The CRRS database comprehensively investigated villagers’ health awareness including that reflected in villagers’ dietary habits. For instance, there is a question asking respondents “Does your family purchase organic, green, or pollution-free vegetables?” and “Does your family purchase organic, green, or pollution-free pork?.” We used these two questions as the proxy variables of health awareness, namely Vegetable and Pork . The optional responses are “No” and “Yes” which are assigned values of 0 and 1, respectively. Table 5 shows the results of the mediating effect analysis using Vegetable and Pork as the mediating variables. The *p* values of both mediating variables are below 0.05, indicating the existence of mediating effects. The Sobel test results suggest that both mediating effects are partial effects: the mediating effect of Vegetable is about 5.12% of the total effect, and the mediating effect of Pork is about 5.47%. Overall, both of the mediating effects of health awareness account for around 5% of the total effects of income inequality on villagers’ health.

**Table 5:** Analysis of mediating effects.

Variables	Health awareness effect (vegetable)			Health awareness effect (pork)		
	(1)	(2)	(3)	(4)	(5)	(6)
	Health	Vegetable	Health	Health	Pork	Health
RD	−0.192^***^ (0.046)	−0.144^***^ (0.027)	−0.182^***^ (0.046)	−0.192^***^ (0.046)	−0.114^***^ (0.025)	−0.181^***^ (0.046)
Vegetable			0.068^**^ (0.028)			
Pork						0.092^***^ (0.030)
ControlVariable	Controlled	Controlled	Controlled	Controlled	Controlled	Controlled
$P>\left|Z\right|$	0.025			0.011		
Mediating Effect	5.12%			5.47%		
Cons	−0.287 (1.283)	−0.785 (0.754)	−0.233 (1.282)	−0.287 (1.283)	0.702 (0.694)	−0.351 (1.282)

Standard errors are in brackets; *, **, *** indicate significant at the significance level of 10, 5, and 1%, respectively.

## 4 Conclusion and policy suggestions

### 4.1 Conclusion

This study seeks to establish an empirical model for examining the impact of income inequality on public health in rural areas of China. Guided by the relative deprivation theory, which suggests that income inequality can result from social comparisons, we calculated the Kakwani index using micro-data from 3,771 individual villagers and 302 village-level samples obtained from the 2019 China Rural Reform and Development Survey (CRRS) database. Utilizing regression techniques, including Ordinary Least Squares (OLS), Ordered Logit (Ologit), and Ordered Probit (Oprobit) models, we conducted our analysis. Our findings reveal a significant negative correlation between income inequality among villagers and their health status. Specifically, villagers with lower income inequality tend to exhibit better health, emphasizing the potential health benefits of reducing income disparities. In our heterogeneity analysis, we considered villagers’ exercise habits and education levels. We found that villagers engaging in regular exercise tend to maintain better health, mitigating the adverse effects of disadvantaged income status. Additionally, education emerged as another avenue to mitigate the negative impact of income inequality on public health. Well-educated villagers typically possess greater knowledge and awareness of health management, buffering the negative effects of income inequality. Through a mediation analysis, we explored the underlying mechanisms through which income inequality influences health. Our analysis revealed that income inequality exerts a discernible influence on villagers’ health awareness, subsequently shaping their overall health status.

This study contributes to addressing a research gap in the intersection of income inequality and public health within the context of China’s rural revitalization strategy. The research findings, illustrating a negative correlation between income inequality and public health as perceived by China’s villagers, provide valuable scientific insights applicable to the implementation of the UN’s SDGs. Furthermore, this study stands as a foundational reference for future inquiries into the current state of public health and its determining factors in rural China. Nevertheless, there are avenues for potential improvement. Firstly, the utilization of the CRRS database is constrained by having only one published data edition, limiting the study to cross-sectional data. Plans are in place to track future releases of the CRRS, enabling the extension of research results accordingly. Secondly, the current CRRS questionnaire items on public health are observed to be general and lacking scientific indices for measuring villagers’ objective health conditions. Future research endeavors aim to explore the possibility of integrating other valuable databases to address this gap.

### 4.2 Policy suggestions

Attaining integrated sustainability within China’s rural revitalization strategy necessitates a comprehensive grasp of the rural population’s needs. This study explores the potential influence of economic advancement through rural revitalization on the broader national health agenda, highlighting health challenges in rural areas. The findings provide valuable insights for policymakers and governments striving to improve public health in China’s rural regions, ultimately fostering integrated sustainability in the country’s rural revitalization efforts. Building upon these findings, we put forth two sets of suggestions:

In terms of national policies, although the Chinese government achieved the significant milestone of eradicating absolute poverty by 2020, enhancing residents’ absolute individual income and living standards, particularly for the rural populace, remains a pivotal task. Policymakers might consider prioritizing the addressing of wealth distribution in society, recognizing it as a fundamental prerequisite for achieving sustainable economic development. Beyond economic considerations, China’s forthcoming rural revitalization strategy should institute comprehensive metrics in public health, focusing on crafting indicators pertinent to the evolving needs of the vast rural population. These indicators would serve as prerequisites for monitoring the progress of the rural revitalization strategy and facilitating necessary policy adjustments.

In terms of practical attempts, a primary focus is directing increased investment towards enhancing public health infrastructures, including healthcare services and other health facilities. Equally paramount is the widespread dissemination of health-related information. Promoting healthy lifestyle choices, such as regular exercise and a balanced diet, holds considerable potential to elevate individuals’ health awareness and, consequently, improve health outcomes. Furthermore, enhancing educational resources in rural areas emerges as a strategic avenue for advancing public health. The correlation between higher incomes and heightened health awareness is intricately linked to the level of education received. Therefore, an earnest effort to elevate educational standards in rural areas not only empowers minds but also serves as a catalyst for broader improvements in health awareness and overall well-being.

## Data availability statement

Publicly available datasets were analyzed in this study. This data can be found at: http://rdi.cass.cn/ggl/202210/t20221024_5551642.shtml.

## Author contributions

JL: Conceptualization, Data curation, Formal analysis, Funding acquisition, Methodology, Supervision, Writing – original draft, Writing – review & editing. KG: Conceptualization, Data curation, Formal analysis, Methodology, Supervision, Writing – original draft, Writing – review & editing. CC: Conceptualization, Data curation, Formal analysis, Funding acquisition, Methodology, Supervision, Writing – original draft, Writing – review & editing.
